# Prospective evaluation of surgical treatment of liver metastasizing pancreatic cancer - ScanPan study protocol

**DOI:** 10.1186/s12893-025-02983-w

**Published:** 2025-07-19

**Authors:** Kristina Hasselgren, Caroline Williamsson, Johanna Wennerblom, Poya Ghorbani, Maria Gustafsson Liljefors, Heikki Huhta, Margareta Heby, Christopher Månsson, Mia I. Johansson, Nils O. Elander, Minna Nortunen, Raija Kallio, Asif Halimi, Daniel Öhlund, Per Sandström, Ernesto Sparrelid, Bergthor Björnsson

**Affiliations:** 1 Division of Surgery, Department of Biomedical and Clinical Sciences, Linköping University, Linköping, Sweden; 2 Department of Surgery, Clinical Sciences Lund, Lund University and Skåne University Hospital, Lund, Sweden; 3 Department of Surgery, The Institute of Clinical Sciences, Sahlgrenska Academy, University of Gothenburg, Gothenburg, Sweden; 4 Division of Surgery and Oncology, Department of Clinical Science, Intervention and Technology, Karolinska Institutet, Karolinska University Hospital, Stockholm, Sweden; 5 Surgery Research Unit, Medical Research Center Oulu, Oulu University Hospital, University of Oulu, Oulu, Finland; 6 Department of Clinical Sciences Lund, Division of Oncology and Pathology, Lund University, Skåne University Hospital, Lund, Sweden; 7 Department of Surgical Sciences, Uppsala University, Uppsala, Sweden; 8 Department of oncology, The Institute of Clinical Sciences, Sahlgrenska Academy, University of Gothenburg, Gothenburg, Sweden; 9 Department of Oncology and Haematology, Oulu University Hospital, University of Oulu, Oulu, Finland; 10 Department of Oncology, Department of Biomedical and Clinical Sciences, Linköping University, Linköping, Sweden; 11 Department of Diagnostics and Intervention, Surgery, Umeå University, Umeå, Linköping, Sweden; 12 Department of Diagnostics and Intervention and Wallenberg Centre for Molecular Medicine, Umeå University (WCMM), Umeå, Sweden; 13 Department of Surgery, Linköping University Hospital, Linköping, Sweden

**Keywords:** Pancreatic cancer, Liver metastases, Resection, Ablation

## Abstract

**Introduction:**

Patients with pancreatic ductal adenocarcinoma (PDAC) have a dismal prognosis. The majority of patients are diagnosed at an advanced stage, and for these patients, the only possible treatment is palliative chemotherapy. There are increasing data from retrospective studies indicating that a subgroup of patients with liver-limited metastases may benefit from surgical treatment of liver metastases. However, there is a need for prospective trials.

**Objective:**

The aim of this study is to prospectively investigate the safety and feasibility of surgically treating patients who are resectable, including those with borderline venous resectable, histopathologically confirmed PDAC, and histopathologically or radiologically confirmed liver metastases.

**Methods:**

Five Swedish and one Finnish hepatopancreaticobiliary (HPB) centre will participate. Eligible patients will be identified at regional multidisciplinary conferences (MDTs). Before inclusion, they will undergo computed tomography (CT), magnetic resonance imaging (MRI, ) and (positron emission tomography computed tomography)PET-CT to rule out extrahepatic metastases. To be included, patients will have to have four or fewer liver metastases, which must be no larger than 5 cm for patients planning for resection and no larger than 2 cm for patients planning for ablation. The metastases may be either synchronous or metachronous. Patients will undergo four months of chemotherapy before surgical treatment (either resection or ablation), and postoperatively, they will undergo two months of chemotherapy. For those with synchronous metastases, resection of the pancreatic tumour will be performed. Follow-up will be performed over two years postoperatively with regular CT scans and assessments of quality of life.

**Conclusions:**

In conclusion, this trial will provide increased knowledge concerning whether surgical treatment of liver metastases from pancreatic cancer can result in improved survival.

**Clinical Trial Number:**

Clinical.Trials.gov (NCT05271110), registered February 26^th^ 2022

**Supplementary Information:**

The online version contains supplementary material available at 10.1186/s12893-025-02983-w.

## Introduction

### Background and rationale

Pancreatic cancer has an increasing incidence, partly due to changes in lifestyle factors, which can result in an increased frequency of obesity and diabetes, but also due to an increase in lifespan [1].

The overall survival for patients with all stages of pancreatic cancer is barely 4 months [2]. Nearly 60% of patients have metastatic disease at the time of diagnosis and may be considered for palliative chemotherapy [2].

The data indicate that surgical treatment of liver metastases can result in improved survival compared with palliative chemotherapy only. In previous studies, which included patients with synchronous as well as metachronous liver metastases, the median survival after resection of liver metastases ranged from 12 to 37 months [3–5], whereas it was approximately 6 months for those treated with palliative chemotherapy [2].

There are also data indicating that survival may be improved for those who undergo surgical treatment for liver metastases in combination with systemic chemotherapy, especially those who respond to chemotherapy [6].

The outcome after surgical treatment seems to be comparable whether the liver metastases are treated with resection or ablation. Ablation of liver metastases can result in a median survival of 14 months [7].

Thus, the available data suggest that the outcome may be improved with a combination of surgical treatment and systemic chemotherapy for patients with pancreatic cancer and liver metastases. Previous studies are, however, retrospective, include rather few patients, and the number of patients undergoing chemotherapy varies.

Therefore, a prospective study is needed to investigate the safety and feasibility of such an approach.

The manuscript complies with the Standard Protocol Items: Recommendations for Interventional Trials (SPIRIT) guidelines.

### Design

This study is a Scandinavian multicentre study in which five Swedish and one Finnish hepatobiliary centre participated. The primary objective of this study was to prospectively investigate the safety, feasibility, and clinical outcome of surgical treatment for liver metastases in patients with pancreatic ductal adenocarcinoma (PDAC). The study will be analysed on an intention-to-treat basis. The presence of a pancreatic tumour will be histopathologically confirmed on biopsies from the primary tumour. Liver metastases will be confirmed via biopsy or assessed via radiology. Tumour markers will be measured and may guide diagnosis. The number of liver metastases accepted for inclusion will be up to four and up to five cm for those planned to undergo resection and up to two cm for those planned to undergo ablation. The metastases may be synchronous or metachronous. Eligible patients will be identified at regional multidisciplinary conferences (MDTs), which are held weekly at each participating centre. For enrolment, the patient must be assessed as suitable for chemotherapy as well as surgical treatment.

If the patient meets the inclusion criteria and accepts participation in the study, the patient will undergo at least four months of chemotherapy before surgical treatment. The chemotherapy regimen is not dictated in the study protocol but is based on national and international recommendations and is ultimately decided upon by the medical oncologist treating the patient. A radiological evaluation will be performed after two months. If radiological progression is evident at the first evaluation change in chemotherapy regimen is recommended. After an additional two months of chemotherapy, a renewed radiological evaluation will be performed. For those with stable disease or response according to the Response Evaluation Criteria in Solid Tumours (RECIST) criteria after four months of chemotherapy [8], resection of the primary tumour, if not already resected, ablation or resection of the liver metastases will be performed, provided that the patient is suitable for surgery. Blood samples, including tumour markers, will be taken at inclusion, at radiological evaluation and during the follow-up. Blood samples are subjected to routine haematological, renal, and liver function tests, and CA 19 − 9 as a tumour marker.

For those with synchronous liver metastases, the resection of the primary tumour and surgical treatment of the liver metastases may be synchronous or sequential. The patients whose liver metastases are planned to be resected will primarily undergo liver- and pancreatic resection at the same procedure. For those whose metastases will be ablated, the metastases will primarily be ablated before the pancreatic resection.

Those with a progressive disease of either the primary tumour and/or the liver metastases, as well as those with deterioration of the clinical condition, will be assessed as palliative and may continue with either chemotherapy, but with a palliative intent or receive best supporting care.

For those with no response, chemotherapy will be continued, and radiological re-evaluation will be performed every other 2 months, as long as the patient tolerates the treatment and the therapy is clinically indicated.

**Fig. 1:**
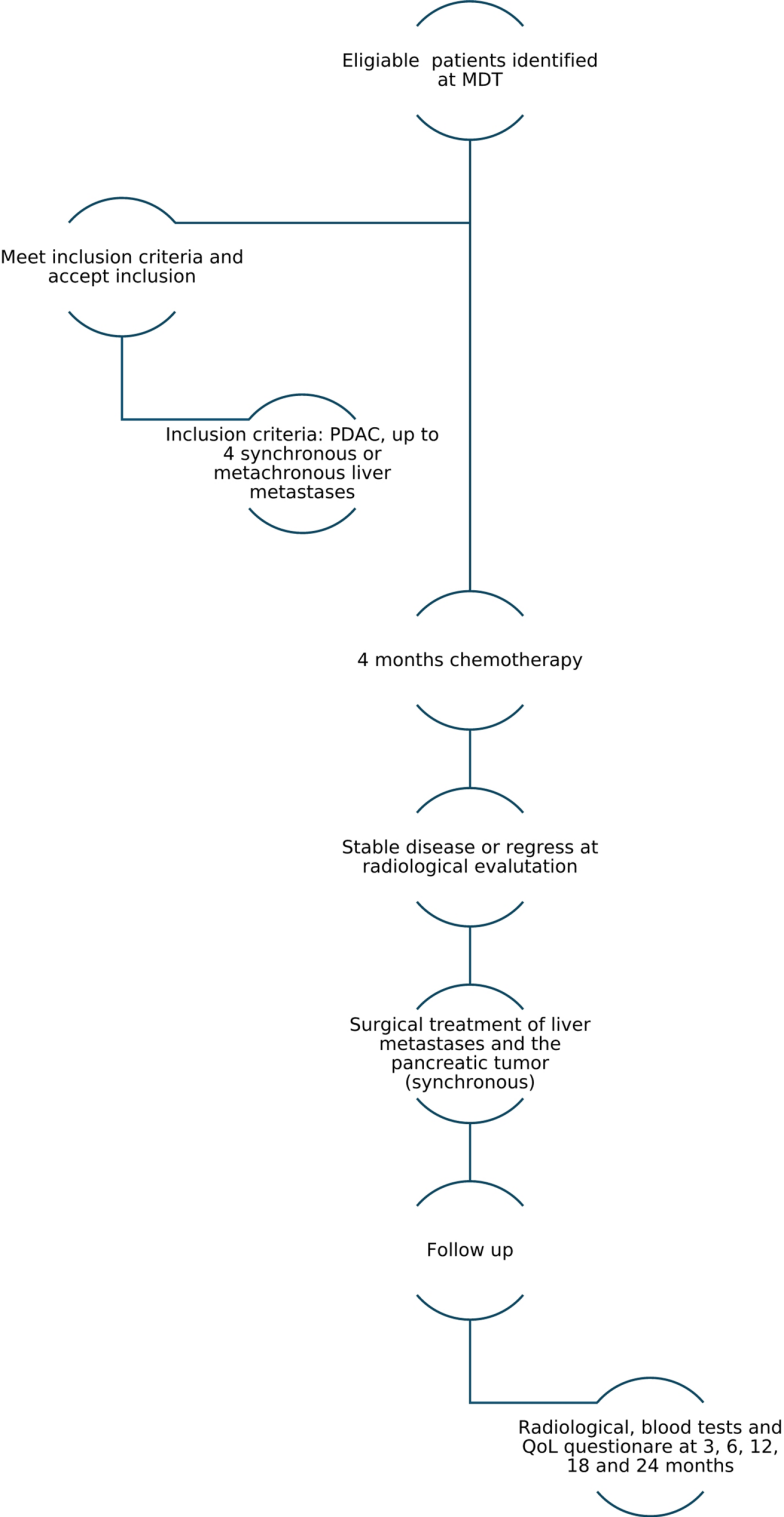
A schematic description of the study, from the identification of eligliable patients at MDT to the end of follow at 24 months post operatively

The inclusion time is planned to be 36 months, from the inclusion of the first patient. Each patient will be followed for 24 months after resection/ablation. The total study time is estimated to be 60 months, from the inclusion of the first patient to the end of follow-up of the last included patient. A flow chart describes the study schematically (Fig. 1).

### Characterization of the participants

The study population will consist of adult patients with resectable or previously resected PDAC with synchronous or metachronous liver metastases.

#### Inclusion criteria

- Age ≥ 18 years.
- Eastern Cooperative Oncology Group (ECOG) score of 0–1.
- The ability to understand verbal and written information and provide verbal and written consent.
- Resectable, including venous borderline resectable, histopathologically confirmed PDAC.
  - Endoscopic ultrasound (EUS)-guided biopsy of the primary tumour is recommended.
  - Percutaneous biopsy of the liver metastases is accepted.
  - If pathological sampling fails, radiological diagnosis combined with elevated tumour marker levels is accepted for diagnosis.
- Up to 4 liver metastases are resectable with an adequate volume of the future liver remnant without volume expansion of the future liver remnant.
  - Synchronous or metachronous liver metastases.
    - Synchronous metastasis was defined as metastasis within 6 months of diagnosis of the primary tumour.
  - Up to 5 cm for metastases planned for resection.
  - Up to 2 cm for metastases planned for ablation.

#### Exclusion criteria

- Progression of tumour (RECIST 1.1) during or after chemotherapy.
- Distant metastases outside the liver.
- Other malignancies within 2 years of inclusion in the study.
- Major nonpancreatic surgery within 4 weeks prior to the inclusion in the study, or a lack of complete recovery from the effects of major surgery.
- Significant concomitant diseases prevent the safe administration of chemotherapy.
- Severe comorbidity.
- Predefined need for arterial reconstruction to resect the primary tumour.
- Pregnancy.

### Treatment

Eligible patients will, before inclusion, undergo CT of the chest and abdomen, MRI of the liver, and PET-CT. Thereafter, a discussion will be held at the regional MDT, and a decision will be made if the patient is a candidate for inclusion. After 2 months of chemotherapy, a radiological evaluation will be performed, and for those with regress or stable disease, an additional 2 months of chemotherapy will be administered, resulting in a total of 4 months of chemotherapy preoperatively. Before surgical treatment, a new radiological evaluation will be performed.

The chemotherapy regimen is not defined in the protocol but is based on national and international guidelines. The most common regimens are modified FOLFIRINOX or Gemcitabine-nab-Paclitaxel. The oncologist at each participating centre ultimately decides the appropriate regimen. For each included patient, the aim is to undergo a total of 6 months of chemotherapy: 4 months before surgery and 2 months after surgery. The chemotherapy regimen may be modified or changed preoperatively and may differ postoperatively.

Toxicity is graded according to the “Common Terminology Criteria for Adverse Events” version 5, and dose reduction is accepted, if indicated.

The chemotherapy may be terminated in advance, either because of toxicity or other medical conditions or because of patient requests. In those cases, the patient is excluded from surgical intervention.

The surgical technique is not predefined; rather, it is performed according to clinical practice at each participating centre and is assessed as suitable for achieving radical resection. Pancreatic resection may be open, laparoscopic, or robotic. Liver metastases may be treated with resection (open, laparoscopic or robotic) or ablation (radiofrequency; RF or microwave; MW), according to clinical assessment and standard practice at each participating centre. Percutaneous biopsy may be performed in the same procedure as ablation. Staging laparoscopy before any surgical procedure is accepted.

Postoperative care is performed according to routine clinical practice. Postoperative complications are registered and graded according to the Clavien‒Dindo classification [9] and the comprehensive complication index (CCI) [10].

### Posttreatment

Each patient will be followed up at 1, 3, 6, 12, 18, and 24 months after surgery, from which the resection renders the patient tumour free. In the follow-up clinical assessment, blood samples, including tumour markers, as well as an assessment of quality of life (QoL) with the EQ-5D questionnaire, are included. CT of the chest and abdomen will be performed 3, 6, 12, 18, and 24 months after treatment for the liver metastases. For those with suspected recurrence, an MRI and/or PET-CT will be performed. Recurrence is diagnosed based on distinct radiological findings. The date of recurrence is noted in the case report form (CRF).

### Statistical analysis

#### Sample size

A total of 64 patients will be included. The sample size calculated is based on the goal of achieving radical resection in the liver in 30% of the included patients. To prove this with a (b ≤ 0.05; 1-b (power): 0.95), the Phase II Gehan’s Method Two Stage Design was used.

#### Analysis plan

The main study parameter is the prevalence of eligible patients, namely, the number of patients with limited disease identified by the MDT.

The primary endpoints are the safety, tolerability, and feasibility of the surgical treatment, which will be analysed in an intention-to-treat manner.

The secondary endpoints are surgical morbidity, radical resection/ablation of liver metastases, radical resection of pancreatic tumours, overall survival, disease-free survival, progression-free survival, and quality of life.

Surgical morbidity will be registered according to the Clavien‒Dindo classification [9] and the Comprehensive Complication Index [10], as well as pancreatic-specific complications. Postoperative pancreatic fistula, postpancreatectomy haemorrhage, and delayed gastric emptying are specific pancreatic complications.

Radical resection in the liver is defined as no evidence of a tumour at the resection line. For those treated with ablation of liver metastases, radical resection will show no evident tumour on CT one month after ablation. Radical resection of the pancreatic tumour will be defined as a ≥ 1 mm margin on all resected surfaces.

Overall survival will be defined as survival from inclusion in the study until death or the end of follow-up.

Disease-free survival will be defined as the time from the last surgical procedure rendering the patient tumour free until evident tumour recurrence (radiological or histopathological proven).

Quality of life will be assessed with the EQ-5D.

The Kaplan‒Meier method will be used to evaluate disease-free survival (DFS) and overall survival (OS) and their median values. DFS and OS will be calculated from the date of inclusion in the study until evidence of recurrent disease, death, or the end of follow-up.

A Cox model will be constructed to evaluate possible factors, including whether the liver metastases are synchronous or metachronous, affecting survival. Otherwise, descriptive statistics will be used.

#### Data collection and management

Data, including electronic CRF, will be collected using the Castor platform.

## Discussion

Despite the low incidence of pancreatic cancer compared with other malignancies that cause a significant healthcare burden, PDAC remains a common cause of cancer-related death. The main reasons for this are the high proportion of patients with disease dissemination combined with relatively limited treatment options. However, in light of new emerging treatment options with T-cell immunotherapy for PDAC, as exemplified by a recent publication in the New England Journal of Medicine, investigating the possibility of applying surgical treatment in settings in which such treatment is currently considered nonadvantageous is important [11].

To our knowledge, this is the first prospective study of the surgical treatment of patients with PDAC and liver metastases. Retrospective studies indicate increased survival for those who undergo resection or ablation of liver metastases [3–7]. This study is an international, multicentre study, with all participating centres being HBP centres, which is a strength; thus, all participating surgeons, medical oncologists, and radiologists have vast experience with this category of patients.

One limitation of the study is the lack of comparator group, and a randomized controlled trial would likely allow for a firmer conclusion regarding the eventual benefit of surgical treatment of liver metastases. One the other hand, this is to our knowledge the first prospective study for this category of patients and therefore this study is also exploring the feasibility of including them in prospective studies.

Another limitation of the study is that for those that will undergo ablation and not resection of liver metastases, no histopathological examination of the lesions may be performed. Therefore, it cannot be ruled out that some patients have benign lesions in the liver, especially those with stable disease at radiological evaluation. The risk is minimized because the protocol dictates that all patients will undergo MRI before inclusion and as evaluation. MRI has high diagnostic accuracy, and small lesions can be detected, especially if gadoxetic acid is used as a contrast agent and if the hepatobiliary phase is included in the examination [12, 13]. A disadvantage of MRI is that the examination may be more challenging to the patient than CT is and that the cost of the examination is greater. The high diagnostic accuracy of MRI, combined with the low risk of complications and fast recovery, motivates ablation as a fully accepted treatment for liver metastases [14].

It could be argued that a disadvantage in the protocol is that the chemotherapy is not specified in the protocol. However, the study was not designed to investigate the response to specific chemotherapy regimens. With this design, it is possible that more patients may continue the stipulated time of preoperative chemotherapy. Furthermore, it may, to a greater degree, reflect the clinical situation in which patients may not tolerate more aggressive chemotherapies, such as FOLFIRINOX.

With respect to QoL, it is important that a more aggressive treatment strategy does not result in an impaired QoL compared with palliative chemotherapy. For patients undergoing palliative chemotherapy for advanced PDAC, the QoL is not improved but rather stabilized at the baseline level [15]. For patients undergoing pancreatic surgery, there is a decline in QoL, but it returns to baseline 6 months postoperatively [16], and the same is shown for patients undergoing resection of colorectal liver metastases [17, 18]. It is reasonable to assume that QoL after resection of liver metastases from PDAC can return to baseline.

Compared with established palliative chemotherapy, including QoL assessment in this study is important because it involves more aggressive treatment.

In conclusion, this trial will provide increased knowledge on whether surgical treatment of liver metastases from pancreatic cancer can improve survival.

## Electronic supplementary material

Below is the link to the electronic supplementary material.

Supplementary Material 1

## Data Availability

No datasets were generated or analysed during the current study.
